# Translation, Cross-Cultural Adaptation, and Psychometric Validation of the TeamSTEPPS^®^ Teamwork Attitudes Questionnaire: A Methodological Study

**DOI:** 10.3390/nursrep16010026

**Published:** 2026-01-15

**Authors:** Leonor Velez, Patrícia Costa, Ana Rita Figueiredo, Mafalda Inácio, Paulo Cruchinho, Elisabete Nunes, Pedro Lucas

**Affiliations:** 1 Nursing Research Innovation and Development Centre of Lisbon (CIDNUR), School of Nursing, University of Lisbon, Avenida Prof. Egas Moniz, 1600-190 Lisbon, Portugal; leonorvelez@campus.esel.pt (L.V.); patriciacosta@esel.pt (P.C.); anaritafigueiredo@esel.pt (A.R.F.); pjcruchinho@esel.pt (P.C.); enunes@esel.pt (E.N.); prlucas@esel.pt (P.L.); 2 Nursing Administration Department, School of Nursing, University of Lisbon, Avenida Prof. Egas Moniz, 1600-190 Lisbon, Portugal; 3 Department of General Surgery and Gastroenterology, Instituto Português de Oncologia de Lisbon, Rua Professor Lima Bastos, 1099-023 Lisbon, Portugal

**Keywords:** interprofessional education, nursing administration research, patient safety, psychometric properties, TeamSTEPPS^®^, validation studies

## Abstract

**Background:** Teamwork and effective communication are widely recognized as essential pillars for the safety and quality of healthcare. However, in Portugal, no validated instrument had previously been available to assess healthcare professionals’ attitudes toward teamwork. This study aimed to translate, culturally adapt, and validate the TeamSTEPPS^®^ Teamwork Attitudes Questionnaire (T-TAQ) for the Portuguese context, resulting in the Portuguese version of the instrument. **Methods:** A methodological study with a quantitative approach was developed. The translation and cultural adaptation process followed internationally recognized guidelines. The sample consisted of 162 healthcare professionals (136 nurses and 26 physicians) from a hospital in Lisbon. Exploratory and confirmatory factor analysis techniques were used to assess construct validity. The internal consistency of the scale was analyzed using Cronbach’s alpha coefficient. **Results:** The Portuguese version comprises 30 items distributed across five dimensions: Effective Leadership Support, Team Functional Performance, Teamwork Coordination, Willingness to Engage in Teamwork, and Team Functioning Supervision. The scale demonstrated a total explained variance of 53.9% and an overall internal consistency coefficient (α) of 0.86, indicating good reliability. Confirmatory factor analysis supported the five-factor structure of the scale (χ^2^/df = 1.461; CFI = 0.900; GFI = 0.821; RMSEA = 0.054; MECVI = 4.731). **Conclusions:** The T-TAQ-PT proved to be a valid, reliable, and robust instrument for assessing healthcare professionals’ individual attitudes toward teamwork, contributing to the development of research and clinical practice in the Portuguese context.

## 1. Introduction

Teamwork and effective communication are widely recognized as fundamental pillars of patient safety and healthcare quality [1,2,3,4,5,6]. The delivery of healthcare requires effective coordination among different professionals, who perform interdependent roles and share common goals oriented toward safety and health outcomes [7,8]. In this context, nurses play a fundamental role as coordinators and leaders of care, influencing continuity of care, interprofessional collaboration, and the creation of safe and sustainable practice environments [9,10,11,12,13,14]. Recent evidence has further demonstrated that teamwork effectiveness is strengthened when teams operate with shared situational awareness and proactive information sharing, which support safer decision-making and coordinated action in complex clinical environments [6,15,16,17]. Interprofessional teamwork contributes not only to clinical coordination but also to the development of resilient working environments, in which team members collectively adapt to uncertainty, negotiate clinical priorities, and sustain safe care under changing conditions, reinforcing teamwork as a protective factor in patient safety performance [15,18]. When team members share common goals, clearly defined roles, and supportive leadership structures, collaboration becomes more consistent and communication more explicit, which enhances collective responsibility for patient outcomes and strengthens the safety culture [6,16]. Teams with stronger collaborative climates and structured communication routines demonstrate higher consistency in care performance, reduced variability in clinical practice, and improved alignment between individual actions and shared patient safety goals, supporting teamwork as a central mechanism for sustaining high-quality and safety-oriented care [6].

Scientific evidence has demonstrated that failures in communication, teamwork, and leadership are directly associated with adverse events, preventable errors, and a reduction in the quality of care [2,3,6,7,19,20,21]. In this regard, effective communication, coordinated teamwork, and leadership have been identified as effective strategies for achieving safe and high-quality care [2,3,21,22,23]. Teamwork should be carried out through interdisciplinary cooperation, as it strengthens communication and coordination among team members [24]. Interprofessional teamwork has increasingly been identified as a central approach to addressing the growing complexity of healthcare, promoting shared decision-making, person-centered care, continuity of care, and enhanced clinical safety [1,7,8]. Communication and collaborative team climates contribute to improved consistency of care and patient safety outcomes, particularly when teams engage in mutual support and collective monitoring of the clinical situation [6,16,18].

The effectiveness of teamwork depends, however, not only on the implementation of organizational structures or training programs but also on healthcare professionals’ individual attitudes toward teamwork [4,25]. Attitudes directly influence collaborative behaviors, communication, clinical decision-making, and compliance to safety practices and are recognized as a key determinant of safety culture and team performance [2,4,5,6,25,26]. Positive teamwork attitudes are associated with greater willingness to speak up, stronger engagement in risk prevention, and the development of supportive team cultures that sustain safety-oriented behaviors in daily practice [16,18,27].

Within this framework, the Team Strategies and Tools to Enhance Performance and Patient Safety (TeamSTEPPS^®^) program was developed by the Agency for Healthcare Research and Quality (AHRQ), in collaboration with the Department of Defense, as a structured response to the need to improve healthcare team performance, following the Institute of Medicine report To Err Is Human, which highlighted the impact of preventable errors on patient safety [22,28]. TeamSTEPPS^®^ is recognized as an international reference for the promotion of patient safety, healthcare quality and efficiency, and cultural change within healthcare organizations [4,22,25,28,29].

The TeamSTEPPS^®^ conceptual model operationalizes teamwork through five dimensions (leadership, team structure, mutual support, situation monitoring and communication), considered core and trainable competencies for effective healthcare team performance [22,28]. These dimensions reflect an integrated combination of knowledge, skills, and attitudes that support collaborative behaviors, adaptation to dynamic clinical situations, and the development of a safety culture within healthcare organizations [2,4,22,25,28]. Additionally, these dimensions are widely recognized in the literature as pillars of team performance, patient safety and healthcare quality [2,4,23,25].

Despite the relevance of this construct, no instrument is currently available in Portugal to assess healthcare professionals’ individual attitudes toward teamwork. This absence represents an important gap in research and clinical practice, as it limits the evaluation of the impact of teamwork training programs, the planning of evidence-based organizational interventions, and the monitoring of safety culture within healthcare institutions [7,25]. Thus, given the importance and relevance of this phenomenon, it was considered essential to undertake the cross-cultural adaptation and validation of the TeamSTEPPS^®^ Teamwork Attitudes Questionnaire (T-TAQ), originally developed by the AHRQ in 2008 and subsequently validated by Baker et al. [22] in 2010. This instrument aims to assess healthcare professionals’ attitudes toward the five teamwork dimensions defined in the TeamSTEPPS^®^ model.

The T-TAQ has been validated and applied in different international contexts, including Sweden and France, where it has been administered to multiprofessional teams [25,29], and in Norway, where it has been validated among nursing students, demonstrating adequate psychometric properties [2]. The cross-cultural adaptation and psychometric validation of measurement instruments are essential processes to ensure conceptual, semantic, and cultural equivalence, as well as the validity and reliability of the results obtained in different sociocultural contexts [30,31]. The validation and cross-cultural adaptation of the T-TAQ for the Portuguese population constitute a relevant milestone for both research and clinical practice at the national level.

Thus, the availability of this tool, appropriately adapted to the Portuguese sociocultural context, will enable the assessment and promotion of collaborative competencies within healthcare teams, support evidence-based training interventions, and foster an organizational culture oriented toward patient safety and quality of care [4,6]. By filling an existing scientific and practical gap, this study provides a substantial contribution to the advancement of interprofessional practice and to the consolidation of a more efficient, cohesive, and person-centered healthcare system. In this way, it contributes to the enhancement of research, nursing management, and clinical practice within the Portuguese context.

This study aimed to translate, cross-culturally adapt, and validate the T-TAQ, resulting in the Portuguese version of this instrument. The following research question was defined: Does the T-TAQ demonstrate adequate content validity, construct validity, concurrent validity, and reliability for the Portuguese population?

## 2. Materials and Methods

### 2.1. Design

A methodological study was developed [32], with a quantitative approach.

The Guidelines for Reporting Reliability and Agreement Studies (GRRAS) were followed [33].

### 2.2. Target Population, Sample, and Sampling Technique

The target population consisted of nurses and physicians who worked at a hospital in the Greater Lisbon area. A convenience sample were used to recruit potential participants. The following selection criteria were defined: being a physician or a nurse, with or without management responsibilities; being a physician or a nurse working in any unit or service of the host institution, including inpatient services, outpatient clinics, emergency departments, delivery rooms, operating rooms, special diagnostic services, and intensive care units. The exclusion criterion was not being a physician or a nurse.

The achieved sample, obtained through a non-probabilistic sampling technique [32], consisted of 136 nurses and 26 physicians. The adequacy of the sample size was evaluated in accordance with methodological recommendations for scale validation, which suggest that acceptable ratios typically range from five to ten participants per item [34,35]. Other authors propose similar practical guidelines, indicating minimum ranges between three and ten respondents per item [36] or optimal intervals of five to fifteen participants [37]. Also, the achieved sample obtained was considered adequate in light of the model structure, as MacCallum et al. [38,39] argue that sample size requirements in factor analysis depend primarily on communality levels and factor over-determination rather than on fixed numerical rules.

### 2.3. Data Collection Instrument

In 2006, the AHRQ, in collaboration with the United States Department of Defense, developed the TeamSTEPPS^®^ program, a team training program aimed at improving teamwork effectiveness and patient safety [28,40]. The T-TAQ questionnaire was developed in 2008 by the AHRQ [40] and validated by Baker et al. [22] in 2010. The objective was to assess individual attitudes toward five dimensions of teamwork: leadership, team structure, mutual support, situation monitoring, and communication. The questionnaire consists of 30 items organized on a five-point frequency Likert-type scale (1. “strongly disagree”; 2. “disagree”; 3. “neither agree nor disagree”; 4. “agree”; 5. “strongly agree”).

### 2.4. Translation and Cross-Cultural Adaptation Process

The translation and cross-cultural adaptation process of the T-TAQ followed the methodological guidelines proposed by Cruchinho et al. [41], Beaton et al. [30,42] and Sousa and Rojjanasrirat [43] and Boateng et al. [44]. As demonstrated by Boateng et al. [44], such frameworks provide structured phases and best-practice steps that support rigorous assessment of content validity, construct validity, reliability and overall methodological quality.

The first stage began with the translation of the instrument from English into Portuguese. The translation was carried out by two independent bilingual and bicultural translators. One had knowledge of the healthcare field, and the other specialized in semantic equivalence.

In the second stage, a synthesis of the translations was conducted through an expert panel meeting. This resulted in a consensus version (synthesized version), which constituted the first Portuguese version of the instrument.

In the third stage, back-translation from Portuguese into English was performed, producing two versions of the instrument. This procedure was conducted by two independent translators, also bilingual and bicultural but without training in the healthcare field. It was verified that all items, when translated back into English, maintained the same meaning as the original instrument.

In the fourth stage, based on expert analysis, the translated and back-translated versions were compared to examine possible ambiguities and discrepancies. No discrepancies were found between versions. The presence of semantic, idiomatic, and conceptual equivalence was concluded, resulting in the pre-final version of the instrument.

In the fifth stage, a pre-test of the pre-final version of the instrument was conducted with 11 participants. No difficulties in comprehension were identified by the participants, nor were any ambiguities reported regarding the content, wording, or format of the items, which demonstrated and confirmed the clarity and adequacy of the instrument.

Thus, the translated and adapted version of the instrument was produced.

### 2.5. Data Collection

Data collection, conducted through the Google Forms^®^ platform, took place between February 2025 and April 2025. It was structured in two sections:

- Sociodemographic characterization, with questions related to age, gender, academic qualifications, professional category, length of professional practice as a nurse or physician, and length of professional practice in the current service;
- Translated and adapted version of the Portuguese version of the scale.

### 2.6. Data Analysis

The data collected was analyzed and processed using IBM^®^ SPSS Statistics^®^, version 22.0, for Windows (Armonk, NY, USA). Confirmatory factor analysis of the questionnaire was performed using IBM^®^ SPSS^®^ AMOS software (version 29; Armonk, NY, USA)), in accordance with the guidelines of Marôco [45].

An exploratory factor analysis (EFA) was first conducted. Prior to extraction, sampling adequacy was verified using the Kaiser–Meyer–Olkin (KMO) index and Bartlett’s test of sphericity. Principal axis factoring was used as the extraction method, and varimax rotation was applied. The criteria for factor retention included eigenvalues greater than 1 and inspection of the scree plot [44,45,46]. Items with factor loadings below 0.30 were examined and considered for removal.

Following the EFA, a confirmatory factor analysis (CFA) was performed. Model fit was assessed using multiple goodness-of-fit indices, including the χ^2^/df ratio, Comparative Fit Index (CFI), Tucker–Lewis Index (TLI), Root Mean Square Error of Approximation (RMSEA), and Standardized Root Mean Square Residual (SRMR), following recommended cut-off values [46].

Convergent validity was examined through the Average Variance Extracted (AVE) and standardized factor loadings, with AVE ≥ 0.50 and loadings ≥0.50 considered acceptable. Internal consistency was evaluated using Cronbach’s alpha and composite reliability for each factor.

### 2.7. Ethical Considerations

The present study was previously approved by the Research Ethics Committee of the host institution on 20 February 2025 (Official Letter No. 30/CE). Participation in the study required the obtaining of free and informed consent from each participant, which ensured information regarding the purpose of the study, data anonymity, maintenance of confidentiality, and the voluntary nature of participation. The conduct of the study fully complied with the ethical principles established in the Declaration of Helsinki [47].

## 3. Results

This section presents the results of the study, including sample characteristics and the psychometric evaluation of the Portuguese version of the T-TAQ. The overall response rate was 23.14%. All respondents who accessed the questionnaire completed all items, resulting in a complete dataset with no missing values.

### 3.1. Sociodemographic Characteristics of the Sample

In the sample consisting of 162 healthcare professionals (136 nurses and 26 physicians), 84% were female. These results are consistent with national data, since according to the Portuguese Board of Nurses [48], 82.8% of nurses were female, and according to the Portuguese Medical Association [49], 58.09% of the medical workforce is female.

The age groups with the highest concentration of professionals were 22–26 years (26.5%) and 37–41 years (21%), which reinforces the presence of a bimodal distribution. The mean age was 36.2 years. This distribution differs from the national distribution, since according to the Portuguese Board of Nurses [48], most nurses (16.97%) were aged between 36 and 40 years, and according to the Portuguese Medical Association [49], 26.7% of physicians were over 65 years of age (an age group not represented in the present study). Regarding academic qualifications, most professionals held a bachelor’s degree, corresponding to 64.2% of the total sample. None of the participants held a short-cycle degree, and only one participant held a doctoral degree (0.6%). In addition, 35.2% of the professionals held a master’s degree. According to the Portuguese Board of Nurses [48], most professionals hold only a bachelor’s degree (74.5%). It was not possible to identify national statistical data regarding the academic qualifications of the medical community.

Regarding the professional category of the sample, approximately 84% of the sample consisted of nurses and 16.1% of physicians. Within the nursing profession, 46.3% were general care nurses, 30.9% were specialist nurses, and 6.8% performed functions as nurse managers. National data indicate that 70.6% of Portuguese nurses are general care nurses, and only 2.2% have advanced competencies in management [48]. Overall, the present data are consistent with national statistics. Regarding the medical staff, 5.6% were resident physicians, 8% were attending physicians, and 2.5% were senior physicians. According to national data on the medical workforce [49], 33.7% of physicians are not specialists. In this sense, there was a predominance of nursing professionals in the study sample.

Considering the length of professional practice as a nurse or physician, most professionals had been practicing for less than 10 years (44.4%). In contrast, 21% of the sample had between 20 and 29 years of experience, and only 4.1% of professionals had been practicing for more than 30 years. These results indicate that the sample is mainly composed of nurses and physicians with shorter professional experience, reflecting a relatively young professional profile.

According to the data presented, most professionals (76.5%) have been working in the current service for less than 10 years.

### 3.2. Psychometric Properties Analysis

#### 3.2.1. Reliability

The internal consistency of the translated and adapted version of the T-TAQ was assessed using Cronbach’s alpha coefficient, yielding a value of 0.86. According to Vilelas [35] this value indicates good internal consistency. In this sense, the instrument presents a good level of reliability and can measure what it is intended to assess.

Internal consistency by dimension was also high: Effective Leadership Support with α = 0.85; Team Functional Performance with α = 0.78; Teamwork Coordination with α = 0.76; Willingness to Engage in Teamwork with α = 0.85; and Team Functioning Supervision with α = 0.79.

#### 3.2.2. Content Validity

The adaptation of the questionnaire to the Portuguese cultural context was carried out to ensure the comprehensibility and relevance of its items for the target population, physicians and nurses [41]. Idiomatic expressions, cultural values, and differences between cultures were taken into consideration to maintain the content validity of the T-TAQ [41].

The content validity of the items and the scale was analyzed by calculating the I-CVI (Item Content Validity Index) and S-CVI (Scale Content Validity Index). Eight experts were used for this analysis, in accordance with Collucci & Milani [50].

The I-CVI per item ranged from 0.875 to 1, values considered adequate according to Coluci e Milani [50]. The S-CVI of the scale was 0.975, a value that indicates excellent content validity, according to Coluci e Milani [50]. The experts used relevance, clarity, representativeness, and cultural relevance as decision criteria.

#### 3.2.3. Construct Validity

Construct validity was analyzed in two complementary phases, both conducted using the same dataset: exploratory factor analysis and confirmatory factor analysis.

- Exploratory factor analysis

The factor structure of the translated and adapted version of the T-TAQ was explored based on the 30 items of the translated and adapted version.

Scree plot analysis was used to define the dimensions of the translated and adapted T-TAQ questionnaire. Factors with eigenvalues equal to or greater than one were considered.

The first approach to exploratory factor analysis was conducted without imposing any constraints on the number of factors. In this initial phase, the first exploratory factor analysis, with an eigenvalue of 1.031, resulted in eight factors that explained approximately 65.198% of the total explained variance. The first approach to exploratory factor analysis was conducted without any constraints on the number of factors.

However, the eight-factor solution showed limited theoretical interpretability, with some factors presenting weak conceptual coherence and a reduced number of items with salient factor loadings. Given that the T-TAQ is a theory-driven instrument with a well-established five-factor structure grounded in the TeamSTEPPS^®^ conceptual framework, a subsequent exploratory factor analysis was conducted specifying a five-factor solution. This approach is recommended in cross-cultural validation studies to assess the adequacy of the original dimensional structure while preserving conceptual equivalence with the source instrument. Based on this rationale, factor rotation was forced, reducing the number of dimensions as the reliability of each factor was evaluated, until a stable final version of the instrument with five factors was achieved. With a five-factor solution and an eigenvalue of 1.406, a total explained variance of 53.907% was obtained.

The reliability of each factor was calculated, as described in Table 1. The dimension with the lowest α is the third dimension. Table 1 presents an interpretation of the internal consistency of each dimension, according to what is described by Vilelas [35].

In order not to exclude items that could distort the constructs intended to be measured by the scale, a factor loading cutoff of 0.30 was established. According to Leech et al. [51] a cut-off point ≥ 0.30 for item communalities is considered good. The translated and validated version of the T-TAQ managed to retain 30 items and five dimensions, as in the original scale. Through Table 1, it is possible to understand the composition of the questionnaire and the nomenclature of each dimension.

After analysis of the rotated component factor matrix, five dimensions were obtained. Each dimension contains variables with different factor loadings, as shown in the following table.

Exploratory factor analysis resulted in the extraction of five factors, which explain 53.907% of the total variance explained, as mentioned previously:

- Effective Leadership Support: Included eight items (item 7 “It is important for leaders to share information with team members”; item 8 “Leaders should create opportunities for information sharing with team members”; item 9 “Effective leaders view mistakes as meaningful learning opportunities”; item 10 “It is the leader’s responsibility to model and influence appropriate team behavior”; item 11 “It is important that leaders devote time to discussing plans for each patient”; item 12 “Leaders should ensure that team members help one another when necessary”; item 13 “Individuals can be taught to analyze the context of situations in order to detect relevant cues”; and item 16 “It is important to monitor the emotional and physical state of team members”). This dimension assesses the ability of the nurse leader to provide effective support to the team, promoting cohesion, communication, and professional well-being, to strengthen trust and effectiveness in teamwork.
- Team Functional Performance: Included five items (item 22 “Offering help to a colleague is an effective strategy for improving team performance”; item 23 “It is appropriate to continue voicing a concern about patient safety until one is sure it has been heard”; item 27 “Adverse events can be reduced by maintaining information sharing with patients and their families”; item 28 “I prefer to work with team members who ask for clarification about the information I provide”; and item 29 “It is important to use a standardized method to communicate information during transitions of care (shift handover, unit transfer, hospital transfer and transfers between services)”). This factor encompasses the assessment of the team’s functional performance, considering how professionals interact, collaborate, and coordinate efforts in achieving common goals.
- Teamwork Coordination: Included seven items (item 1 “It is important to ask patients and their families for feedback about their care experience”; item 2 “Patients are fundamental members of the care team”; item 3 “The institution’s administration influences the success of care delivery teams”; item 6 “High-performing healthcare teams share common characteristics with high-performing teams in other sectors”; item 15 “Even staff who are not part of the direct care team should be encouraged to look for and report changes in a patient’s condition”; item 18 “Team members who monitor and manage their own emotional and physical state at work are more effective”; and item 19 “To be effective, team members should understand their colleagues’ working conditions”). This dimension reflects the team’s ability to coordinate efforts and articulate tasks to achieve common objectives, ensuring a balanced distribution of responsibilities, information sharing, and the promotion of a collaborative environment and mutual support. The leader is represented as a facilitator of this process by ensuring fair task distribution and fostering mutual assistance and attentiveness to others.
- Willingness to Engage in Teamwork: Included four items (item 20 “Asking a team member for help is a sign of being unable to perform one’s work adequately”; item 21 “Offering help to team members is a sign that one does not have enough work to do”; item 24 “Conflicts between team members do not affect patient safety”; and item 30 “It is almost impossible to train team members to become effective communicators”). This factor refers to professionals’ willingness to contribute to teamwork by developing communication skills, understanding their own role and those of colleagues, reducing interprofessional barriers, and benefiting from leader support, thereby enhancing team cohesion and performance.
- Team Functioning Supervision: Included six items (item 4 “A team’s mission takes precedence over the individual goals of each member”; item 5 “Effective team members anticipate the needs of other team members”; item 17 “When a team member is tired or anxious, it is appropriate to offer assistance”; item 25 “Teams that do not communicate effectively significantly increase their risk of making errors”; item 26 “Ineffective communication is the most common cause of reported errors”; and item 14 “Regular monitoring of patients is fundamental for effective team performance”). This factor refers to the role of the leader as the main authority in monitoring the achievement of objectives, identifying needs to be addressed, and verifying communication processes among professionals, ensuring team guidance, motivation, and empowerment, as well as the effective achievement of collective and individual outcomes.

Concluding this process, the final structure of the translated and adapted version of the scale was defined, which was designated as T-TAQ-PT. The high explained variance and robust reliability coefficients reinforce the validity of the model and the adequacy of the instrument to the Portuguese cultural context.

- Confirmatory factor analysis

**Figure 1:**
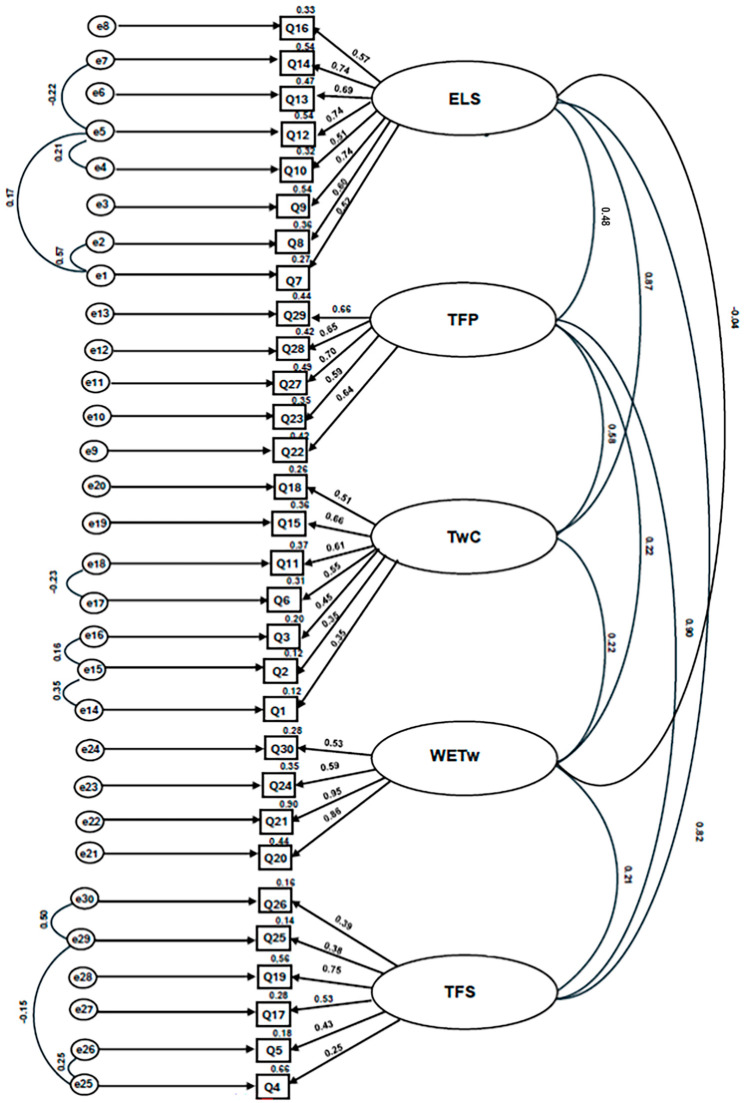
Pentafactorial Model of the Portuguese Version of the T-TAQ. Q1–Q30 represent the observable variables (questionnaire items); e1-e25 pairs of covariances; Q1 = It is important to ask patients and their families for feedback about their care experience; Q2 = Patients are fundamental members of the care team; Q3 = The institution’s administration influences the success of care delivery teams; Q4 = A team’s mission takes precedence over the individual goals of each member; Q5 = Effective team members anticipate the needs of other team members; Q6 = High-performing healthcare teams share common characteristics with high-performing teams in other sectors; Q7 = It is important for leaders to share information with team members; Q8 = Leaders should create opportunities for information sharing with team members; Q9 = Effective leaders view mistakes as meaningful learning opportunities; Q10 = It is the leader’s responsibility to model and influence appropriate team behavior; Q11 = It is important that leaders devote time to discussing plans for each patient; Q12 = Leaders should ensure that team members help one another when necessary; Q13 = Individuals can be taught to analyze the context of situations in order to detect relevant cues; Q14 = Regular monitoring of patients is fundamental for effective team performance; Q15 = Even staff who are not part of the direct care team should be encouraged to look for and report changes in a patient’s condition; Q16 = It is important to monitor the emotional and physical state of team members; Q17 = When a team member is tired or anxious, it is appropriate to offer assistance; Q18 = Team members who monitor and manage their own emotional and physical state at work are more effective; Q19 = To be effective, team members should understand their colleagues’ working conditions; Q20 = Asking a team member for help is a sign of being unable to perform one’s work adequately; Q21 = Offering help to team members is a sign that one does not have enough work to do; Q22 = Offering help to a colleague is an effective strategy for improving team performance; Q23 = It is appropriate to continue voicing a concern about patient safety until one is sure it has been heard; Q24 = Conflicts between team members do not affect patient safety; Q25 = Teams that do not communicate effectively significantly increase their risk of making errors; Q26 = Ineffective communication is the most common cause of reported errors; Q27 = Adverse events can be reduced by maintaining information sharing with patients and their families; Q28 = I prefer to work with team members who ask for clarification about the information I provide; Q29 = It is important to use a standardized method to communicate information during transitions of care; Q30 = It is almost impossible to train team members to become effective communicators.

The pentafactorial structure of the T-TAQ-PT was evaluated using confirmatory factor analysis, performed with IBM^®^ SPSS^®^ AMOS software (version 29), in accordance with the guidelines of Marôco [45].
The normality of variables was assessed according to univariate and multivariate skewness (Sk) and kurtosis (Ku) coefficients. No severe violations of normal distribution were observed, as all variables showed Sk values below three and Ku values below ten [46]. To ensure the quality of the data under analysis, the presence of outliers was examined using the squared Mahalanobis distance (D^2^).
The overall model fit quality was analyzed using several indices, which are considered improvable due to being marginal [45]. To adjust the model, pairs of error covariances between items were analyzed. The following pairs of covariances were corrected, with the following modification indices: e1-e2 (50.195), e1-e5 (6.738), e4-e5 (6.508), e5-e7 (7.032); e14-e15 (20.888), e15-e16 (5.840), e17-e18 (6.006); e23-e24 (25.373); e25-e26 (10.219), e25-e29 (6.333), e29-e30 (40.997). According to Marôco [45] these corrections should be made up to a value of 6 in the covariance modification indices. The minimum value we obtained was 5.840, which we still corrected because it was very close to 6 and to obtain better values in the adjustment of the final factorial model. Thus, eleven covariance corrections were performed, resulting in an improvement in the global fit indices. An overall acceptable model fit was obtained, with adequate incremental fit indices and marginal absolute fit indices: CMIN/DF =1.461 (≤3); RMR = 0.044 (≤0.05); GFI = 0.821 (≥0.90); AGFI = 0.783 (≥0.90); Delta2 = 0.903 (≥0.90); CFI = 0.900 (≥0.90); SRMR = 0.0748 (≤0.08); RMSEA = 0.054 (≤0.05) e MECVI = 4.731 (<5).
Most items in the factor model have standardized factor loadings (λ) greater than 0.5. Thus, the final factor model demonstrated a good/very good quality of fit.
The following figure (Figure 1) presents the final T-TAQ-PT model with standardized factor loadings and corrected covariances. The obtained configuration reflects a stable factor structure, confirming the robustness of the proposed model for application in the Portuguese population of health professionals.

## 4. Discussion

The translation and cross-cultural adaptation of the T-TAQ to the Portuguese context were carried out in accordance with a complex and rigorous process, following the recommendations defined by Cruchinho et al. [41], Beaton et al. [30,42] and Sousa and Rojjanasrirat [43]. The methodological process followed a systematic and rigorous approach, to ensure not only translation fidelity but also the cultural and linguistic relevance of the items for the Portuguese population. The sociocultural specificities of the context were taken into consideration, which contributed to the clarity of the items, ensuring the content validity of the adapted version. The adaptation of the questionnaire to the Portuguese cultural context was performed to guarantee the comprehensibility and relevance of its items for the target population, physicians and nurses [41]. Idiomatic expressions, cultural values, and differences between cultures were considered to maintain the content validity of the T-TAQ [41]. As a result, the Portuguese version of the instrument proved to be conceptually equivalent to the original, maintaining all items with their integrity and relevance in relation to the construct assessed.

The analysis of the psychometric properties of the instrument was conducted based on a methodologically rigorous process, focusing on a sample of 162 healthcare professionals (nurses and physicians).

The Teamwork Attitudes Scale obtained a reliability value with a Cronbach’s alpha of 0.86. It presents good internal consistency [35], thus being able to measure what it is intended to assess. In contrast, the original questionnaire developed by Baker et al. [22] did not calculate the alpha for the total scale. The original article of the scale only reports that the total alpha is >0.7 (reasonable consistency). Likewise, Diep et al. [29] did not calculate the overall internal consistency of the instrument. The Swedish and Norwegian cross-cultural adaptations are the only ones that also evaluated this parameter. The Norwegian study obtained a total scale alpha of 0.79, and the Swedish study obtained a total scale alpha of 0.70. Both revealed reasonable internal consistency [35]. Thus, it is concluded that the T-TAQ-PT presents a Cronbach’s alpha value higher than the other cross-cultural adaptations. Therefore, it demonstrated higher internal consistency than the Norwegian and Swedish studies.

Initially, exploratory factor analysis was conducted without any imposed constraints, resulting in an eight-factor solution. To achieve a structure consistent with the original T-TAQ structure and the other cross-cultural adaptations, factor rotation was forced until an instrument composed of five factors was achieved (through factor rotation and reduction in the number of dimensions as the reliability of each factor was evaluated). Although the five-dimensional structure was maintained, the names assigned to each dimension differ from those of the other versions of the T-TAQ (original and cross-cultural adaptations) (Table 2). A reorganization of items by dimension was also observed. These modifications did not influence the reliability of the model. Thus, the T-TAQ-PT instrument allows for the development of international comparative studies while always respecting the sociocultural particularities of the Portuguese cultural context.

Regarding confirmatory factor analysis, the results evidenced the stability of the pentafactorial structure of the T-TAQ-PT, demonstrating an acceptable model fit to the study population, with the following values: CMIN/DF = 1.461; RMR = 0.044; GFI = 0.821; AGFI = 0.783; Delta2 = 0.903; CFI = 0.900; SRMR = 0.0748; RMSEA = 0.054; and MECVI = 4.731.

These indices fall within the parameters recommended by Marôco [45], providing a consistent empirical basis for the validity of the proposed structure in the Portuguese context.

In the original study developed by Baker et al. [22], the authors did not disclose the values related to model fit indices. Therefore, it was not possible to compare the model fit indices of the T-TAQ-PT with the original instrument.

On the other hand, it was possible to compare these values with previously developed cross-cultural adaptations. The Swedish version [25] classified the model fit as acceptable (CMIN/DF of 2.96, RMSEA of 0.068, and CFI of 0.808). The results of the French version [29] indicated good model fit (CMIN/DF = 1.65, CFI = 0.925, SRMR = 0.060, and RMSEA = 0.033). The Norwegian version [52] reported acceptable fit values for two indices (RMSEA = 0.043; CMIN/DF = 1.862) and values below the reference thresholds for one index (CFI = 0.832).

The differences found between the Portuguese model, the original model, and the three cross-cultural adaptations already developed may reflect cultural and contextual discrepancies in the way Portuguese professionals, physicians and nurses, value teamwork and its components. The factor model presented five dimensions, which reorganize the content of the original instrument in a semantically coherent manner and in accordance with the Portuguese professional and sociocultural reality.

It was thus concluded that the acceptable adequacy of the fit indices obtained through confirmatory factor analysis demonstrated the usefulness of this new, reliable, and relevant instrument for assessing individual attitudes toward teamwork, specifically in the context of nursing and medicine in Portugal. Accordingly, the reliability and validity of the instrument, in its Portuguese version, are confirmed for the advancement of research, as well as for the professional practice of healthcare professionals.

### 4.1. Implications for Practice

The T-TAQ-PT is an instrument that allows for the assessment of healthcare professionals’ individual attitudes toward teamwork, an essential component for achieving safe care. Thus, the T-TAQ-PT is a fundamental tool for the development of management and research domains in the healthcare field.

This new instrument, valid and reliable for assessing individual attitudes toward teamwork, presents an innovative character, stimulates the emergence of new scientific publications, encourages adherence to team training programs, allows for the evaluation of teams, identifies gaps in practice to be addressed, and values teamwork concepts (such as mutual support, leadership, the care recipient, interprofessional teams, and intraprofessional teams).

The T-TAQ-PT proves to be a necessary instrument for improving the safety and quality of care provided in Portugal, as it values essential aspects for the establishment and development of effective teamwork. Furthermore, although it has been validated for the healthcare population, it may be applied by researchers whose objective is solely to study individual attitudes toward teamwork or even be used to evaluate other team training programs, such as TeamSTEPPS^®^ [22].

### 4.2. Limitations

First, it should be noted that the recruitment of professionals, physicians and nurses, was carried out in a single healthcare institution, using a non-probabilistic sampling method, which may generate sampling bias. However, the inclusion of different hospital services, thus adopting heterogeneity in clinical practice contexts, resulted in outcomes representative of the reality of Portuguese nurses and physicians who use the Teamwork Attitudes Scale.

Cultural differences between Portugal and the other countries where the T-TAQ was cross-culturally adapted may constitute another limitation of the study. The assessment of individual attitudes toward teamwork may differ according to organizational culture, the structure of healthcare systems, teamworking practices, and different leadership approaches within a given team. Thus, the Portuguese questionnaire demonstrates professional and sociocultural specificities of the Portuguese cultural context.

In this sense, differences in the form and implementation of teamwork training across different cultural contexts may also represent a limitation to the translation and validation of the T-TAQ. Divergences in training, due to cultural differences, alter the way each professional understands and values attitudes toward teamwork. This may influence the comparability of the Portuguese results with other cross-cultural adaptations.

## 5. Conclusions

The translation and validation study of the T-TAQ for the Portuguese cultural context provides an instrument to Portuguese healthcare organizations for use in team training and in the assessment of individual attitudes toward teamwork. The T-TAQ-PT is a tool that allows Portuguese managers to evaluate healthcare professionals’ attitudes regarding teamwork.

The instrument presents good results in terms of validity and reliability. Consequently, it is a valid and reliable instrument for measuring individual attitudes toward teamwork. Thus, it may be applied in future studies aimed at diagnosing existing attitudes toward teamwork within a system, unit, or hospital. All Portuguese healthcare contexts will benefit from the application of this scale, as it allows for the identification of gaps in care practice and the improvement of performance. In fact, it may also be used by any researcher who wishes to measure these attitudes in other contexts.

In summary, the T-TAQ-PT is a tool that promotes the improvement of nursing and medical care while also enhancing organizational systems themselves.

## Figures and Tables

**Table 1:** Final factor structure: Distribution of items by factor, factor loadings, and Cronbach’s alpha coefficients.

		Dimensions				
Total Items	Items	Effective Leadership Support	Team Functional Performance	Teamwork Coordination	Willingness to Engage in Teamwork	Team Functioning Supervision
8	7	0.828	0.021	−0.084	−0.003	0.069
	8	0.769	0.106	0.036	−0.060	0.146
	9	0.649	0.056	0.415	−0.090	0.093
	10	0.496	0.270	0.405	0.010	−0.240
	12	0.727	0.060	0.391	0.024	−0.134
	13	0.533	0.161	0.388	0.068	0.161
	14	0.543	0.214	0.397	−0.040	0.211
	16	0.552	0.246	0.099	−0.140	0.294
5	22	0.163	0.701	−0.027	−0.077	0.261
	23	0.355	0.565	−0.083	0.269	0.113
	27	0.139	0.668	0.133	0.133	0.055
	28	0.103	0.644	0.148	0.067	0.106
	29	0.033	0.763	0.147	−0.050	0.034
7	1	0.200	0.055	0.478	−0.239	−0.016
	2	0.264	0.050	0.323	−0.173	0294
	3	0.043	0.060	0.694	−0.134	0.118
	6	0.108	0.175	0.569	0.072	0.296
	11	0.411	0.181	0.447	0.160	−0.061
	15	0.310	0.147	0.550	0.129	0.100
	18	0.036	0.324	0.450	0.232	0.198
4	20	−0.018	0.110	0.104	0.829	0.015
	21	−0.087	0.123	0.100	0.835	0.070
	24	0.068	0.006	−0.187	0.795	0.093
	30	−0.055	0.021	−0.062	0.744	−0.069
6	4	0.027	−0.031	0.211	0.320	0.613
	5	0.184	0.166	0.315	0.031	0.475
	17	0.363	0.356	0.132	−0.174	0.478
	19	0.154	0.128	0.306	0.136	0.308
	25	0.007	0.157	0.153	−0.004	0.335
	26	−0.011	0.130	0.350	0.166	0.374
Cronbach’s alpha		0.85	0.78	0.76	0.85	0.79
		0.86				

**Table 2:** Comparison of the T-TAQ-PT with other validations and cross-cultural adaptations.

Versions	Domains/Dimensions	No. of Items	Items	Cronbach’s Alpha	
T-TAQ-PT	Effective Leadership Support	8	7, 8, 9, 10, 12, 13, 14, 16	0.85	0.86
	Team Functional Performance	5	22, 23, 27, 28, 29	0.78	
	Teamwork Coordination	7	1, 2, 3, 6, 11, 15, 18	0.76	
	Willingness to Engage in Teamwork	4	20, 21, 24, 30	0.85	
	Team Functioning Supervision	6	4, 5, 17, 19, 25, 26	0.79	
Baker et al. [22]	Team Structure	6	1, 2, 3, 4, 5, 6	0.70	Not calculated
	Leadership	6	7, 8, 9, 10, 11,12	0.81	
	Situation Monitoring	6	13, 14, 15, 16, 17, 18	0.74	
	Mutual Support	6	19, 20, 21, 22, 23, 24	0.41	
	Communication	6	25, 26, 27, 28, 29, 30	0.74	
Hall-Lord et al. [25]	Team Structure	6	1, 2, 3, 4, 5, 6	0.87	0.70
	Leadership	6	7, 8, 9, 10, 11,12	0.87	
	Situation Monitoring	6	13, 14, 15, 16, 17, 18	0.74	
	Mutual Support	6	19, 20, 21, 22, 23, 24	0.41	
	Communication	6	25, 26, 27, 28, 29, 30	0.63	
Diep et al. [29]	Team Structure	6	1, 2, 3, 4, 5, 6	0.75	Not calculated
	Leadership	6	7, 8, 9, 10, 11,12	0.75	
	Situation Monitoring	6	13, 14, 15, 16, 17, 18	0.58	
	Mutual Support	6	19, 20, 21, 22, 23, 24	0.55	
	Communication	6	25, 26, 27, 28, 29, 30	0.72	
Karlsen et al. [52]	Team Structure	6	1, 2, 3, 4, 5, 6	0.46	0.79
	Leadership	6	7, 8, 9, 10, 11,12	0.62	
	Situation Monitoring	6	13, 14, 15, 16, 17, 18	0.7	
	Mutual Support	6	19, 20, 21, 22, 23, 24	0.44	
	Communication	6	25, 26, 27, 28, 29, 30	0.56	

## Data Availability

Restrictions apply to the availability of these data. Data were obtained from a third party and are available with the permission of the third party.

## Public Involvement Statement

No public involvement in any aspect of this research.

## Guidelines and Standards Statement

This manuscript was drafted against the checklist for reporting of studies of reliability and agreement (GRAAS) Statement: Guidelines for Reporting Reliability and Agreement Studies.

## Abbreviations

The following abbreviations are used in this manuscript:

AGFI	Adjusted Goodness of Fit
AHRQ	Agency Healthcare Research Quality
CFI	Comparative Fit Index
CMIN/DF	Chi-Square Minimum Discrepancy/Degrees of Freedom
GFI	Goodness of Fit Index
MECVI	Modified Expected Cross-Validation Index
RMR	Root Mean Square Residual
RMSEA	Root Mean Square Error of Approximation
SRMR	Standardized Root Mean Square Residual
T-TAQ	TeamSTEPPS^®^ Teamwork Attitudes Questionnaire
